# Restriction of MMTV by APOBEC3

**DOI:** 10.1186/1742-4690-8-S2-P89

**Published:** 2011-12-01

**Authors:** Martin Kraase, Constantine Konstantoulas, Stanislav Indik

**Affiliations:** 1 Department für Pathobiologie, Institut für Virologie, Veterinärmedizinische Universität Wien, Vienna, A-1210, Austria

## Background

Host cells have developed a broad range of mechanisms to counteract retroviral infection. Besides Trim5α (tripartite motif-containing protein 5alpha) and Tetherin, APOBEC3 proteins (apolipoprotein B mRNA editing enzyme catalytic polypeptide-like 3), especially APOBEC3G and APOBEC3F, are major factors in the post entry inhibition of retroviruses. These proteins are incorporated into budding virions and exhibit inhibitory activity by interfering with reverse transcription and/or by deamination of cytosine to uracil in the proviral minus strand, resulting in hypermutation of the viral DNA.

Mouse mammary tumor virus, MMTV, is a murine pathogen causing mammary adenocarcinomas and T-cell lymphomas in infected animals. The betaretrovirus, which is transmitted from mother to pubs via milk, infects dendritic cells, T-cells, B-cells and mammary epithelial cells (MEC).

Previous studies showed that MMTV is inhibited by mouse APOBEC3 and human APOBEC3G proteins. However, the virus is also known to replicate in cells expressing APOBEC3 proteins, suggesting a partial resistance to the APOBEC-mediated antiviral effect.

Therefore, we sought to analyse the anti-MMTV activity of APOBEC3 proteins derived from various species and compare it to the antiviral activity imposed on ΔVif HIV-1.

## Material and methods

MMTV- and HIV-1-based infectious particles were prepared by co-transfection of 293T cells with an MMTV- or HIV-1-based indicator construct carrying GFP, together with a plasmid expressing MMTVor HIV-1 Gag and Pol proteins, a vector expressing the VSV-G protein and finally with varied amounts of APOBEC3-expressing plasmids. The antiviral activity of APOBEC3 proteins was determined by measuring the reduction of GFP-positive target cells in the presence of APOBEC3.

## Results

We found MMTV to be approximately 10-fold more resistant to murine APOBEC3Δex5 and human APOBEC3G proteins than ΔVif HIV-1. Furthermore, other APOBEC3 proteins such as APOBEC3G from rhesus macaque and hAPOBEC3F showed only moderate effect on MMTV.

The mechanism responsible for the partial resistance to APOBEC3 proteins is currently unknown. Because we were able to detect the mAPOBEC3 and hAPOBEC3 in virions, a mechanism which involves exclusion of APOBEC3 from virus particles seems unlikely.

Additionally, we investigated the antiviral activity of novel splice variants of mAPOBEC3 identified from PBMCs of C57/BL6 mice. We found these variants to show low activity against both MMTV and ΔVif HIV-1.

## Conclusions

Taken together, our data suggest that MMTV is partially resistant to the antiviral effects of APOBEC3 proteins. We found that expression of APOBEC3G from rhesus macaque or hAPOBEC3F have only moderate effect on MMTV. Furthermore, we observed that MMTV is inhibited by mAPOBEC3Δex5 and hAPOBEC3G, but the extent of inhibition is significantly lower than that detected with ΔVif HIV-1.

